# Capturing affective states in everyday life - Comparing psychometric properties of two affect measures

**DOI:** 10.1016/j.nsa.2026.107026

**Published:** 2026-09-15

**Authors:** Matthias F. Limberger, Lisa M. Störkel, Johanna Hepp, Inga Niedtfeld, Markus Reichert, Philip S. Santangelo, Lisa-Marie Hartnagel, Oksana Berhe, Andreas Meyer-Lindenberg, Heike Tost, Ulrich W. Ebner-Priemer

**Affiliations:** a Mental mHealth Lab, Institute of Sports and Sports Science, Karlsruhe Institute of Technology, Karlsruhe, Germany; b Department of Psychosomatic Medicine and Psychotherapy, Central Institute of Mental Health, Medical Faculty Mannheim, Heidelberg University, Mannheim, Germany; c Department of Sport and Exercise Science, Faculty of Natural and Life Sciences, University of Salzburg, Salzburg, Austria; d Vitos Hospital for Psychiatry and Psychotherapy Giessen-Marburg, Giessen, Germany; e Department of Psychiatry and Psychotherapy, Central Institute of Mental Health, Medical Faculty Mannheim, University of Heidelberg, Mannheim, Germany; f Department of Behavioural and Cognitive Sciences, Luxembourg University, Esch-sur-Alzette, Luxembourg

**Keywords:** Negative affect, e-diary, Psychometric properties, Ambulatory assessment, Ecological momentary assessment, Experience sampling method

## Abstract

E-diary items and scales face three contradicting challenges, originating from their repeated assessment: To achieve high compliance and to limit participant's burden a minimal number of items is aspired, to achieve reliability on a momentary level more items per scale are advantageous. As psychometric properties and distributions of e-diary scales are important but rarely reported, we used a clinical- (N = 51) and a community-based (N = 353) intensive longitudinal dataset comprising the momentary Multidimensional Mood Questionnaire (mMDMQ) and the Positive and Negative Affect Items (PNAI), both measuring affect, to investigate and compare, in an exploratory approach, distribution indices, psychometric properties, and assumptions for multilevel modeling. In the community sample, all scales of the mMDMQ and the positive affect scale of the PNAI showed very good psychometric properties and distributions. In contrast, the negative affect scale of the PNAI showed right-skewness and floor effects on the item and scale level, and even in the residuals of the multilevel models. In the clinical sample, the overall picture was opposite with mMDMQ again showing reasonable distributions, but this time positive affect of the PNAI revealing problematic properties. Carefully selecting momentary scales and reporting their psychometric properties is essential in e-diary research.

Taking real momentum with the invention of smartphones, e-diary methods are nowadays a common method in psychological research, often used under the labels Ecological Momentary Assessment (Stone et al., 2002), Experience Sampling Method (Csikszentmihalyi and Larson, 1987), or Ambulatory Assessment (Fahrenberg et al., 2007). Whereas these methods have well-known advantages in common, like the real-time and the real-world perspective, as well as the possibility to track dynamics over time, they also come with certain challenges, which are often associated with the intensive longitudinal nature of the assessed data.

One specific challenge in e-diary research is the balance between assessment reliability and validity vs. participant burden. As items must be answered multiple times, e-diary studies may impose substantial demands on participants and increase participant burden. High-burden protocols may have negative effects on compliance (Gable et al., 2000; Litt et al., 1998). Besides problems related to missing data, such as unreliable estimates, this may also lead to biased samples of participants, favouring those who are highly motivated, conscientious and agreeable (Scollon et al., 2003). Accordingly, e-diary measures are usually designed as briefly as possible.

Brief scales, however, sacrifice redundancy which is especially useful in improving reliability. In other words, adding additional items that measure the same construct to an existing scale usually increases reliability. Unfortunately, this approach is limited in e-diary studies. Even worse, e-diary studies are often used for studying dynamic processes and change (Trull et al., 2015). For decades, methodologists were concerned that measuring change would lead to unreliable results (e.g., Cronbach and Furby, 1970; Rogosa, 1988). Systematic change variation is often small relative to random measurement error variation and between-subject differences, and this can lead to biased inferences and loss of statistical power (Scherbaum and Pesner, 2019). The usual approach to improving measurement precision in studies of change is to use longer and more elaborate measures, which is impractical in e-diary studies because of the already substantial demands on participants (Bolger et al., 2003; Reis and Gable, 2000). However, reliable measures on a momentary basis are needed to investigate the dynamics of constructs.

Not surprisingly, reporting guidelines on e-diary research requests for reporting psychometric properties and distributions (Stone and Shiffman, 2002). Unfortunately, empirical evidence shows that these requests are not echoed in the scientific community, as Trull and Ebner-Priemer (2020) reported. Only a minority of studies reported psychometric properties, such as reliability, validity, or sensitivity of change, as well as distribution indices and assumptions of used statistical methods (Trull and Ebner-Priemer, 2020). However, having valid and reliable measurements is a central prerequisite for sound conclusions.

Fortunately, the unprecedented development in computational power pushed the development of new and sophisticated statistical analysis methods, especially in the field of intensive longitudinal data. In recent years, new approaches regarding psychometrics in intensive longitudinal data have been developed, including Omega composite reliability, which we refer to as “McDonald's omega” for better readability (Geldhof et al., 2014), the Spearman-Brown-corrected-correlation (Eisinga et al., 2013), and reliability based on variance components (Generalizability Theory framework) (Cranford et al., 2006). Nonetheless, the psychometric properties of scales and their items are still scarcely reported (Trull and Ebner-Priemer, 2020). Even for some scales measuring affect, which is one of the most often assessed construct in e-diary based psychological research, comprehensive information on basic distribution, psychometric properties, and assumptions are not available, yet.

As psychometric properties and distributions of e-diary scales are rarely reported, we used two existing intensive longitudinal datasets comprising two fundamental different but commonly used measures of affect, namely the momentary Multidimensional Mood Questionnaire (mMDMQ) and the Positive and Negative Affect Items (PNAI)*, a heavily modified and abridged forms of PANAS (*Watson et al., 1988*) and PANAS-X (*Watson and Clark, 1994*), respectively*. We investigated and compared, in an exploratory approach, distribution indices, psychometric properties, and assumptions for multilevel modeling. In detail, a) to shed light on distribution issues, we reported distribution indices (like location-, dispersion-, and shape parameters) of the items and scales used and b) calculated the intra-class-correlation-coefficient (*ICC*), the intraindividual standard deviation (*ISD*), and the root mean squares of successive differences (*rMSSD*). To address psychometric properties. c) we examined the reliability of the scales with different approaches (McDonald's Omega, Person-Brown corrected Correlation, Generalabity Theory based reliability), and d) calculated the sensitivity to change, e) we examined the residual distribution of different models with all scales of the two instruments (mMDMQ and PNAI).

## 1 Method

### 1.1 Participants

We included two different samples in the data analyses: a community sample and a clinical sample with nonsuicidal self-injuries (NSSI). The community sample was recruited within the framework of the psychoepidemiologcal center (PEZ) at the Central Institute of Mental Health (CIMH) Mannheim, Germany as part of the URGENCY study (Impact of Urbanicity on Genetics, Cerebral Functioning and Structure and Condition in Young People(Gan et al., 2021; Reichert et al., 2020; Tost et al., 2019)) at the Central Institute of Mental Health in Mannheim (CIMH), funded by the Ministry of Science, Research and the Arts of the State of Baden-Wuerttemberg, Germany (MWK, grant 42-04HV.MED(16)/16/1). The sample was randomly drawn from the local population register from December 2014 to Novenber 2018 within an age range of 18-28 years. Participants with acute diseases, chronic endocrine, cardiovascular, immunological, or clinically manifested mental disorders, and participants of consent or legal incapacity were excluded. The URGENCY study also assessed accelerometric data and fMRI and participants received monetary compensation for their participation. The compensation was up to 200€ depending on the amount of test in which they participated. The final sample comprised 353 participants (57,1% female) with a mean age of 23.0 years (*SD* = 2.8).

The second sample with 51 females with high mental health symptom load was also recruited at the CIMH, as part of a study on Non-Suicidal Self-Injury (Hepp et al., 2021; Störkel et al., 2021, 2023). This study was supported by a Young Investigator Award of the Central Institute of Mental Health Mannheim, awarded to IN , see open methods and open data here (https://osf.io/uqmky). Age ranged from 18 to 45 (*M* = 23.9 years (*SD* = 6.72)). Inclusion criterion was a DSM-5 nonsuicidal self-injury disorder diagnosis, and frequent NSSI events in the past two weeks (more than 2 incidents per week). Comorbid diagnoses were Major depression (n = 33), Dysthymia (n = 4), Substance abuse (n = 2), Somatic pain disorder (n = 1), Anorexia Nervosa (n = 6), Bulimia Nervosa (n = 5), Social phobia (n = 11), Specific phobia (n = 6), Generalized anxiety disorder (n = 2), Panic disorder (n = 6), Agoraphobia without panic (n = 2), Posttraumatic stress disorder (n = 25), Obsessive compulsive disorder (n = 6), Attention deficit disorder (n = 1), and Borderline personality disorder (n = 32). *Exclusion criteria* were*:* lifetime diagnosis of schizophrenic disorders, mental retardation/developmental disorders, substance dependency within the last 6 months, current injuries not related to NSSI (e.g. slipped disc, operation, etc.), BMI < 17.5 or > 34, pregnancy, current use of cannabis or other stimulating drugs, and medication with opiates, naltrexone, cortisone, or opioid analgesics. For detailed Information see (Störkel et al., 2023). Like in the community sample, participants received monetary compensation for their participation.

### 1.2 e-diary procedure

In the *community sample*, participants carried a smartphone (MotorolaMoto G) for seven consecutive days. Before the assessment, participants received an extensive briefing regarding the usage of the smartphone. The study design was a combined protocol, integrating time-based and GPS-triggered assessments, besides triggers at fixed times (08:00 and 22:20). In detail, as the study focused on the influence of the urban environment on mental well-being (Tost et al., 2019), the GPS-based trigger algorithm detected distances larger than 500 m from the last assessment point to trigger new assessments, whereas e-diary assessments were restrained to occur not more often than every 40 min and at least every 100 min.

A smartphone prompted the participants via an acoustic, visual, and vibration signal every 40 to 100 min within the 7:30 to 22:30 period, which resulted in at least nine to a maximum of 22 triggers per day. The participants were offered the chance to postpone an e-diary prompt for a maximum of 15 min. This combined sampling protocol (time- and location-based) to acquire participants’ affect ratings during everyday life and the implementation on the Android phones were programmed using movisensXS, version 0.6.3658 (movisens GmbH, Karlsruhe, Germany, xs.movisens.com).

In the *NSSI sample*, the assessment lasted for 15 consecutive days. Participants got around five semi-randomized prompts per day (min. interval between prompts 2 h) and were able to initiate a prompt by themselves after an actual NSSI event. Additionally, participants responded to control prompts if they reported a high NSSI urge during a radom prompt, but did not engage in NSSI afterwards. NSSI and control reports were followed by three follow-up prompts (after 10, 20 and 30 min). This resulted in an average of 65 random prompt assessments (non-NSSI assessments) per participant.

The study protocols were approved by the Ethical Committee of the Medical Faculty of Heidelberg University, Germany (protocol number 2014-555N-MA for the community sample and 2014-601N-MA for the clinical sample), and were carried out following the Declaration of Helsinki (World Medical Association, 2013). All participants provided written informed consent prior to their inclusion in the study.

### 1.3 e-diary scales and items

In both studies affective states were assessed with two commonly utilized e-diary scales. A six-item scale developed and validated by Wilhelm and Schoebi (2007), designed to assess within-person fluctuations of mood as a three-dimensional construct via e-diaries. Based on the originally German-language Multidimensional Mood Questionnaire (MDMQ) (Steyer et al., 1994), this short scale assesses three basic affective dimensions: *valence*, *energetic arousal*, and *calmness*. Each scale results from two bipolar items with one item formulated from positive to negative and one in the opposite direction. *Valence* is represented by the items *content* to *discontent* (zufrieden-unzufrieden) and *unwell* to *well* (unwohl-wohl). *Energetic arousal* is represented by *full of energy* to *without energy* (energiegeladen-energielos) and *tired* to *awake* (müde-wach). *Calmness* is represented by *relaxed* to *tense* (entspannt-angespannt) and *agitated* to *calm* (unruhig-ruhig). We presented the bipolar items in mixed order and reversed polarity according to Wilhelm and Schoebi (2007) using visual analog scales (0–100). We refer to this instrument as momentary Multidimensional Mood Questionnaire (mMDMQ).

The second e-diary scale for assessing affective states was similar to the Positive and Negative Affect Schedule (PANAS; Watson et al., 1988). Whereas the original PANAS consists of a fixed set of items, most e-diary studies use a similar approach, namely ask for the intensity of a set of positive and negative adjectives, but items themselves vary between studies, research groups, and papers (e.g. Eisele et al., 2021). Accordingly, we refer to such sets of items as the Positive and Negative Affect Items (PNAI), to avoid that the item selection could be mistaken for the validated PANAS. The PNAI of the community sample consist of six positive (*cheerful* (fröhlich), *content* (zufrieden), *energetic* (tatkräftig), *enthusiastic* (enthusiastisch), *relaxed* (entspannt), *happy* (glücklich)) and the nine negative items (*lonely* (einsam), *sad* (traurig), *insecure* (unsicher), *afraid* (ängstlich), *down* (niedergeschlagen), *guilty* (schuldig), *depressed* (deprimiert), *suspicious* (misstrauisch), *irritated* (gereizt)). The PNAI items were answered on a 7-point Likert scale ranging from 1 (not at all) to 7 (very).

For the NSSI sample six positive and eight negative PANAS-X items were used as the second affective states measure (i.e., PNAI). The six positive items were: *daring* (wagemutig), *attentive* (aufmerksam), *delighted* (erfreut), *bold* (kühn), *happy* (glücklich), and *concentrated* (konzentriert). The eight negative items were: *disgusted* (angeekelt), *loathing* (hasserfüllt), *downhearted* (niedergeschlagen), *afraid* (ängstlich), *alone* (allein), *hostile* (feindselig), *nervous* (nervös), and *blameworthy* (tadelnswert). The selected items of the PANAS-X were answered on the 1-5 Likert scale, wereas the mMDMQ items in this sample had to be answered on a 0-6 bipolar visual scale (---, --, - 0, +, ++, +++).

It is to mention, that between the two samples the overlap of identical items is only one for the PA and two for the NA scale. This limits the comparability of the two samples and scales, but as it is not unusual that PANAS-like unipolar affect items differ between studies, data with this kind of item selection are still very valuable to examine potential problems.

### 1.4 Data analyses

Transformation: The two different scales were assessed in the two studies with different response scales (community sample: PNAI: 1-7; mMDMQ: 0-100; NSSI sample: PANAS-X: 1-5; mMDMQ: 0-6). To ensure comparability between the two scales (mMDMQ, PNAI) and the two samples (community, NSSI) at the descriptive level, the PNAI items were transformed in both samples, to a range from 0 to 100. In the community sample, only the PNAI items were transformed (((x-1)/6)*100) to match the range from 0 to 100. In the NSSI sample, the PNAI items were transformed (((x-1)/4)*100) and the mMDMQ items had to be transformed (((x/6)*100)) to the range of 0-100. Importantly, this transformation of values from a range of 0-6 or 1-5, to 0-100 does not change the distribution of the data.

Descriptive Statistics: Starting with descriptive analyses we computed mean, standard deviation, the intraclass correlation coefficient (*ICC*), skewness, and kurtosis on an item and scale level. We calculated for all affect items and scales the intraclass correlation coefficient (*ICC*). The *ICC* was calculated with an two level empty model with only a random intercept for each variable. So the *ICC* relates the between variance to the total variance (*ICC* = between variance/(within- + between variance) and is regarded as the percentage of between variance of the total variance (Singer and Willett, 2003). Practically, items with a higher value show higher between variance and lower within variance.

Standard deviations and successive differences: In a second step, we calculated intraindividual standard deviations (*ISD*) for each item and scale (Eid and Diener, 1999) to capture the variability around each participants individual mean across all measurement time points. Items with higher *ISD*s indicate higher within-participant variability. We also computed the root mean square of successive differences (*rMSSD*) (Ebner-Priemer et al., 2007; Neumann et al., 1941), which constitutes the average squared change in affect from one measurement point to the next per person. We only took changes within the same day into account. The *rMSSD* depicts affective instability within each participant. We calculated all indices for both, items and scales, to investigate if possible skewed distributions on the item level are evened out on the scale level.

Within-/Between variance and variance proportions: In a further step, we decomposed variance into a three-level model, to distinguish variation between persons, within the same person between different days, and within-person within the same day. We analyzed the proportion as well as the total amount of between-person vs. within-person variation on a day and momentary level. To do this, we calculated a three-level empty model with random intercept only and three variance components for every single scale.

Reliabilities: To increase comparability to the literature and comparability across scales, we applied several approaches to calculate reliability, namely McDonald's omega (Geldhof et al., 2014), the Spearman-Brown-corrected-correlation (Eisinga et al., 2013), and reliability indices based on variance components as suggested in generalizability theory (Cranford et al., 2006).

The currently most often used measure of reliability in e-diary research is McDonald's Omega (Geldhof et al., 2014). McDonald's Omega is calculated based on multilevel confirmatory factor analysis (MCFA) which allows separating reliability on the between- and within-person level. At least three items are needed to calculate a MCFA. We calculated McDonald's Omega in R with the package multilevelTools (R package version 4.5.0. https://CRAN.R-project.org/package=multilevelTools). For two-item scales, like the mMDMQ, the best approach to calculate reliability is the Spearman-Brown-corrected correlation (Eisinga et al., 2013).

We also calculated reliability based on generalized theory as demonstrated by Cranford et al. (2006). To calculate generalizability coefficients, we followed Scott et al. (2020), who based their within-subject and their between-subject reliability coefficient on Cranford et al.’s (2006) equation (4) and equation (5), respectively. The within-person reliability coefficient describes the precision of measurement to detect a systematic change from moment to moment within-persons. As Scott et al. (2020), we refer to this coefficient as R_WP_. As a first step, we decomposed variance in all scales into differences between-persons, as well as fluctuations within-persons. Using IBM SPSS Statistics Version 31 (https://www.ibm.com/products/spss), we decomposed the total variance into multiple parts due to person, occasion, item, the interactions of person*item, occasion*item, person*occasion, and error. Error is the residual variance that is not explained by person, occasion, item, and interactions, as mentioned above. In detail, the equation for R_WP_ is as follows:

(1) $$R_{WP}=\frac{\sigma_{p*k}^{2}}{\sigma_{p*k}^{2}+\left[\frac{\sigma_{e}^{2}}{m}\right]}$$

(*p* = person, *k* = occasion (e.g., moment or day), *e* = error, *i* = item, *m* = number of items).

To estimate the reliability of between-person differences across all observations over the entire study, we used R_BP_. R_BP_ was also used by Scott et al. (2020) and originally developed by Cranford et al.’s (2006) equation (5). In this approach, the residual error variance is divided by k*m to account for the fixed number of assessments and items over which the person's average score is computed.

(2) $$R_{BP}=\frac{\sigma_{p}^{2}+\left[\frac{\sigma_{p*i}^{2}}{m}\right]}{\sigma_{p}^{2}+\left[\frac{\sigma_{p*i}^{2}}{m}\right]+\left[\frac{\sigma_{e}^{2}}{km}\right]}$$

(*p* = person, *k* = occasion (e.g., moment or day), *e* = error, *i* = item, *m* = number of items).

Sensitivity to change: Sensitivity to change is another important psychometric property, especially in intensive longitudinal modeling as the main focus of interest is on the dynamics of variables. As there are no standard approaches to calculating sensitivity to change in multilevel models, Wilhelm and Schoebi (2007) suggested to use the ratio of latent within variance to latent between variance as parameter, with higher ratio values indexing higher sensitivity to change.

Distribution of residuals: Finally, we tested the distribution of the residuals for all scales on the within- and between level. we report the values of the skewness as well as the kurtosis for both samples separately. Therefore we calculated two level null models (intercept only) for all scales in both samples. We also examined the residuals for all scales with qq-plots.

With the exception of the variance component analysis, all analyses were performed using R Version 4.5.0 (https://www.R-project.org/).

## 2 Results of the community sample

### 2.1 Items

In the community sample, the 353 participants completed an average of 85 measurements (*SD* = 11.6) with a compliance rate of 79,6% (*SD* = 17.1). Descriptives on single-item characteristics for the PNAI, separated into *NA* items and *PA* items, and mMDMQ items can be found in Table 1. The ratings on single *NA* items were very low in general, suggesting floor effects. In detail, mean item values across situations and participants ranged between 3.02 (*SD* = 11.59) for *guilty* and 11.20 (*SD* = 20.84) for *irritated* on the to 0 to 100 transformed range. The *PA* items revealed more balanced mean values ranging from 46.76 (*SD* = 26.69) for *enthusiastic* to 66.38 (*SD* = 22.87) for *content*. The *mMDMQ* items also revealed reasonable mean values, ranging from 57.89 (*SD* = 23.28) on the *full of energy/without energy* item to 72.69 (*SD* = 20.37) on the *unwell/well* item*.* Concurrently*,* the overall standard deviations, including within and between subject components, of *NA* items were rather low, compared to *PA* items and mMDMQ items which revealed 30% larger overall standard deviations, on average. One cause of the small variance in the *NA* items is the complete lack of within-person variance in several participants. For example, 30.8% of all participants did not report any varying values for *suspicious*. In the *PA* items, in contrast, only *enthusiastic* had no variation within a single individuum, whereas in the mMDMQ items only 1 to 3 participants showed missing within-subject variance on single items.

**Table 1:** Descriptives, including Intraclass Correlation Coefficients, Intraindividual Standard Deviations, and Mean Squared Successive Differences, PNAI and mMDMQ items (Community Sample N = 353).

Items	Mean (SD)	ICC	Skewness	Kurtosis	ISD Mean (SD)	rMSSD Mean (SD)
At the moment I feel … PNAI Items (0-100)^a^						
lonely (einsam)	6.64 (17.39)	0.39	3.23	10.76	11.87 (8.51)	14.14 (9.84)
sad (traurig)	6.26 (16.90)	0.49	3.36	11.76	10.60 (8.00)	12.27 (8.59)
insecure (unsicher)	8.31 (17.85)	0.40	2.59	6.88	12.62 (7.50)	15.82 (9.27)
afraid (ängstlich)	3.72 (12.60)	0.40	4.40	21.66	7.31 (7.55)	8.87 (8.73)
down (niedergeschlagen)	9.01 (19.73)	0.40	2.48	5.74	13.51 (8.68)	15.68 (9.48)
guilty (schuldig)	3.02 (11.59)	0.37	4.97	28.02	6.63 (6.92)	8.16 (8.35)
depressed (deprimiert)	6.65 (17.37)	0.46	3.10	9.69	10.93 (8.48)	13.00 (9.46)
suspicious (misstrauisch)	3.56 (12.71)	0.53	4.67	24.34	6.71 (7.30)	8.50 (8.98)
irritated (gereizt)	11.20 (20.84)	0.33	2.12	4.05	16.03 (7.83)	19.04 (9.21)

cheerful (fröhlich)	65.53 (22.33)	0.40	−0.56	0.20	16.93 (5.21)	18.97 (6.13)
content (zufrieden)	66.38 (22.87)	0.35	−0.70	0.40	18.07 (5.37)	20.83 (6.62)
energetic (tatkräftig)	52.11 (26.13)	0.33	−0.30	−0.49	20.70 (5.99)	23.53 (7.56)
enthusiastic (enthusiastisch)	46.74 (26.69)	0.45	−0.17	−0.63	18.98 (5.90)	21.46 (6.86)
relaxed (entspannt)	62.47 (24.65)	0.30	−0.54	−0.09	20.10 (5.52)	23.73 (6.94)
happy (glücklich)	65.37 (23.25)	0.43	−0.68	0.41	17.11 (5.75)	19.04 (6.67)

mMDMQ Items (0-100)						
agitated/calm *(unruhig/ruhig)*	70.35 (20.85)	0.36	−0.95	0.53	15.99 (6.14)	19.37 (7.66)
tense/relaxed^#^*(angespannt/entspannt*)	67.79 (22.39)	0.30	−0.68	−0.28	18.05 (5.96)	21.12 (7.03)

discontent/content^#^*(unzufrieden/zufrieden)*	70.97 (21.00)	0.31	−0.93	0.49	16.64 (5.96)	19.04 (7.07)
unwell/well *(unwohl/wohl)*	72.69 (20.37)	0.36	−1.07	1.01	15.72 (6.07)	18.01 (7.15)

tired/awake *(müde/wach)*	58.74 (28.33)	0.20	−0.39	−1.08	24.74 (5.91)	25.98 (7.23)
without energy/full of energy^#^*(energielos/energievoll)*	57.89 (23.28)	0.23	−0.30	−0.71	19.91 (5.34)	21.57 (6.41)

Note: PNAI = Positive and Negative Affect Items; mMDMQ = momentary Multidimensional Mood Questionnaire, SD = standard deviation, ICC = intraclass correlation, ISD = intraindividual standard deviation, rMSSD = root mean square of successive differences.

# recoded Variables (100-x), labels adapted to recoded direction.

a PNAI Items were transformed from 1 to 5 Likert-scale to a range of 0-100.

The *ICC* of the PNAI items ranged all 0.30 or above, from 0.30 (*relaxed*) to 0.53 (*suspicious*), while among the mMDMQ items three out of six scored 0.30 or below (*tired/awake* = 0.20; *full of energy/without energy =* 0.23; *relaxed/tense* = 0.30), revealing more within-subject variance. Overall, negative PNAI items did depict the lowest within-subject variance.

Skewness, depicted in the third column of Table 1, describes the symmetry of the distribution of the single items. *NA* items were extremely positively skewed, i.e., right-skewed (min = 2.12, max = 4.97). In contrast, the *PA* items as well as the mMDMQ items scored within acceptable limits regarding normal distribution. *PA* items were all left-skewed (min = −0.70, max = −0.17), as where the mMDMQ items (min = −0.96, max = −0.30).

The kurtosis in the fourth column shows the steepness of a distribution related to the normal distribution. All *NA* items were extremely leptokurtic due to the overrepresentation of the lowest possible value “0”, ranging from 4.05 to 28.02 for the kurtosis. For *PA* items, as well as mMDMQ items, the deviation from the normal distribution was low. The highest deviation from normal, regarding the kurtosis, for the *PA* items was −0.63 (min = −0.63, max = 0.41). The mMDMQ items showed counterbalanced shapes of distribution, as three items showed platykurtic (min = −1.08, max = −0.28) and three items leptokurtic (min = 0.49, max = 1.01) distributions.

As expected, the *ISDs*, the averaged within-subject standard deviations of the *NA* items ranged also quite low from 6.63 to 16.03, especially in comparison to the *PA* items and *mMDMQ* items. The *rMSSD*, i.e. an index that captures changes from moment to moment, of *NA* items showed much lower scores compared to the *rMSSD*s of *PA* items and mMDMQ items. In detail, *NA* items scored from 8.16 (*SD* = 8.35) (*guilty*) to 19.04 (*SD* = 9.21) (*irritated*), whereas *PA* items scored from 18.97 (*SD* = 6.13) (*cheerful*) to 23.53 (*SD* = 7.94) (*relaxed*) and mMDMQ items from 18.01 (*SD* = 7.15) (*unwell/well*) to 25.98 (*SD* = 7.23) (*tired/awake*). Accordingly, *NA* items seem less suited to depict short-term changes in affective states.

### 2.2 Scales

Descriptives on scale characteristics are presented in Table 2, which is important as in psychological research usually scales are used and not items. Aggregating items to scales normally comes with positive effects regarding distribution. However, and similar to our analyses on the item level, the *NA* scale scored with 6.49 (*SD* = 11.54) on a very low level, again suggesting floor effects. The means of the mMDMQ scales, as well as the *PA* scale of the PNAI were in a medium scale range from 71.83 (*SD* = 19.04) for *valence*, 69.07 (*SD* = 19.69) for *calmness*, 58.31 (*SD* = 23.74) for *energetic arousal*, and 59.76 (*SD* = 18.80) for *PA*.

**Table 2:** Means, Intraindividual Standard Deviation, Mean Squared Successive Differences, and Intra Cluster Correlation of PNAI and mMDMQ scales. (Community Sample N = 353).

Scales	Mean (SD)	ICC	Skewness	Kurtosis	ISD Mean (SD)	rMSSD Mean (SD)
PNAI Scales (0-100)^a^						
negative affect	6.49 (11.54)	0.58	3.11	12.30	6.71 (4.69)	7.35 (4.77)
positive affect	59.76 (18.80)	0.47	−0.44	0.20	13.30 (4.23)	13.81 (4.50)

mMDMQ Scales (0-100)						
valence	71.83 (19.04)	0.35	−0.92	0.69	14.67 (5.56)	16.25 (6.42)
calmness	69.07 (19.69)	0.35	−0.74	0.14	15.19 (5.39)	19.07 (6.67)
energetic arousal	58.31 (23.74)	0.21	−0.35	−0.82	20.54 (5.04)	21.12 (5.94)

Note. SD = standard deviation, ISD = intraindividual standard deviation, rMSSD = root mean square of successive differences, ICC = intraclass correlation.

a PNAI Items were transformed from 1 to 5 Likert-scale values to a range of 0-100.

The *ICC* of the PNAI scales with values of 0.47 for the *PA* scale and 0.58 for *NA* scale are both higher than the scales of the mMDMQ (*ranging from* 0.21 to 0.35). In other words, the mMDMQ is much better at depicting within-subject variance as the PNAIs *PA* and *NA* scales. Unfortunately, the skewed distributions of the *NA* items did not sufficiently improve by aggregating the single items to the *NA* scale. In detail, the *NA* scale did show a pronounced skewness of 3.11 and an even more pronounced kurtosis of 12.30, whereas all other scales revealed skewness and kurtosis values smaller than 1.0.

The *ISD* of the *NA* scale (*ISD* = 6.71) was again lowest compared to those of the *PA* scale (13.30) and the three mMDMQ scales, ranging from 14.67 to 20.54. The root mean square of successive differences (*rMSSD*), i.e. short-term changes in affective states, were lowest in the PNAI *NA* scale (*rMSSD* = 7.35; *SD* = 4.77), medium in the PNAI *PA* scale (*rMSSD* = 13.81; *SD* = 4.50), and higher in the mMDMQ scales (*ranging from* 16.25 (*SD* = 6.42) to 21.12 (*SD* = 5.94)). Thus, short-term changes in affective states could be better depicted by the mMDMQ than by the PNAI scales. Due to the linear transformation of the values, with no change in distributions, findings reported on the original scales showed the same effects.

Within-/Between Variance and Variance Proportions: Fig. 1 depicts a three-level variance decomposition for all scales, with the percentage of variance in the left part of the figure and absolute variance in the right part. Looking at the rate, the *NA* scale showed, with 55%, the highest amount of between variance, resulting in 45% remaining within-subject variance (12% within-persons between days and 33% within-persons within days). All the other scales showed higher percentages of within-subject variance. However, 45% within-subject variance still seems reasonable for multilevel analyses.

**Fig. 1:**
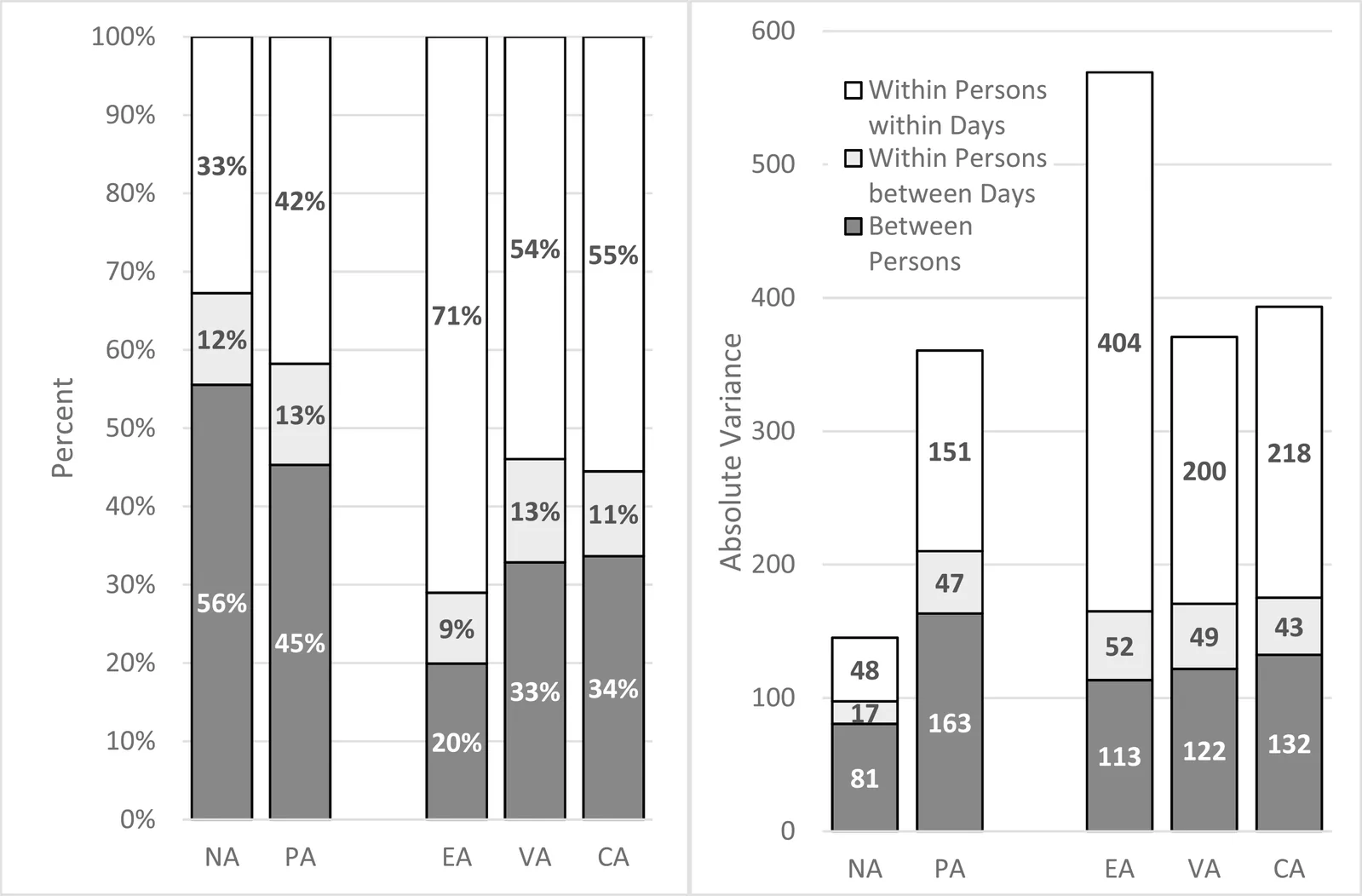
Variance: Within and Between Persons and Days. Proportion and Total Values of the PNAI and mMDMQ scales. (all Scales range from 0 to 100) (Community Sample N = 353) NA = negative affect, PA = positive affect, EA = energetic arousal, VA = valence, CA = calmness.

Regarding the total amount of the variance components, the *NA* scale showed clearly the lowest values in general, whereas the other scales had at least the double amount of total variance. Considering only variance within-persons within days and within-persons between days, the *NA* scale had at least three times less variance than the other scales.

Table 3 reports the reliability of scales on a within- and between-subject level. We present McDonald's Omega for all scales with at least three items (*PA*, *NA*) and the Spearman-Brown corrected correlation for the two-item scales (*valence*, *tense arousal*, *energetic arousal*). In the community sample, The PNAI scales showed acceptable to good reliability on the within-subject level, with an *Omega* of 0.81 for the *PA* scale and an *Omega* of 0.78 for the *NA* scale. The reliability values on the between-level were higher (0.93 for the *PA* scale and 0.95 for *NA* scale). The scales of the mMDMQ achieved also acceptable to good reliability values on the within- (*energetic arousal* = 0.82; *valence* = 0.79; *calmness* = 0.74) and between-subject level (*energetic arousal* = 0.77; *valence* = 0.84; *calmness* = 0.88) for the Spearman-Brown corrected correlation. In the community sample the energetic arousal scale showed a higher value (0.82) on the within person level than on the between person level (0.77) for the Spearman-Brown corrected correlation, this is uncommon. The in general low coefficients for the mMDMQ on the between person level, compared to the NSSI sample, points to a heterogenic group. The reliability estimates based on the generalizability theory (Cranford et al., 2006) which were calculated for the PNAI and mMDMQ scales, achieved all very high values (0.98-0.99) on the between person level, and a range from 0.68 to 0.77 on the within person level. The reliabitiy estimates correspond in general well to the Spearman Brown corrected correlations resp. to McDonald's omega.

**Table 3:** Between- and Within-Person Reliabilities (McDonald's Omega, Spearman-Brown corrected correlations, and Cranford et al., 2006).

		within			between	
	McDonalds Omega	Spearman-Brown	Cranford et al. (2006)	McDonalds Omega	Spearman-Brown	Cranford et al. (2006)
***Community Sample (N = 353)***						
negative affect	0.78		0.77	0.95		0.99
positive affect	0.81		0.73	0.93		0.99


energetic arousal^a^		0.82	0.74		0.77	0.98
valence^a^		0.79	0.75		0.84	0.98
calmness^a^		0.74	0.68		0.88	0.98


***NSSI Sample (N = 51)***						

negative affect	0.77		0.77	0.92		0.99
positive affect	0.74		0.73	0.85		0.99


energetic arousal^a^		0.75	0.74		0.92	0.98
valence^a^		0.75	0.73		0.95	0.98
calmness^a^		0.68	0.69		0.92	0.99

a The MDMQ Scales consist of only 2 items. Therefore, Omega cannot be calculated and the Spearman-Brown corrected correlation is reported instead.

Table 4 depicts sensitivity to change as the ratio of latent within variance to latent between variance, with higher ratio values indexing higher sensitivity to change. All three mMDMQ scales showed a sensitivity of change between 1.26 (*calmness*) and 4.39 (*energetic arousal*). The *PA* scale of the PNAI showed a comparable sensitivity of change ratio (*PA* = 0.98), whereas the sensitivity to changes in *negative affect* was much lower (*NA* = 0.52).

**Table 4:** Latent-, Residual-Variance & Within-/Between-Variance Ratio as Sensitivity to change indicator (PNAI scales transformed to range 0-100) (Community Sample N = 353).

	Latent Variance	Residual Variance	Total Variance	Latent/Total	within/between ratio
Within					
negative affect	47.27	118.79	166.06	0.28	0.52
positive affect	155.09	220.78	375.87	0.41	0.98

energetic arousal	492.55	158.68	651.22	0.76	4.39
valence	204.94	102.83	307.77	0.67	2.06
calmness	156.35	130.91	287.26	0.54	1.26

Between					
negative affect	90.79	36.91	127.70	0.71	
positive affect	158.81	68.93	227.74	0.70	

energetic arousal	112.13	52.95	165.08	0.68	
valence	99.30	38.85	138.14	0.72	
calmness	124.31	39.24	163.55	0.76	

As in multilevel modeling, the normal distribution of the residuals is a prerequisite, but not the normal distribution of items itself, we tested the distribution of the residuals. To do so, we calculated the residuals of the two level null models (intercept only) for all scales. We calculated the residuals for the within-as well as the between level. Skewness and kurtosis of the residuals of all models are reported in Table S1 in the Supplement. The skewness, as well as the kurtosis values of the residuals of the *NA* scale, were higher compared to all other scales. The qq-plots of the different models are also to find in the supplement (Fig. S1 and S2)), with the *NA* scale visually violating the assumption of normality significantly.

## 3 Results of the NSSI sample

### 3.1 Items

In the NSSI sample, 51 participants completed on average 65 non-NSSI measurements (*SD* = 15.6). Table 5 depicts the descriptives on the single-item level of the NSSI sample for the PNAI and mMDMQ items. Mean scores in the *PA-* and the *NA* scale were numerically lower compared to the values of the mMDMQ items. As expected in a clinical sample, the *NA* items showed on a descriptive level in general higher scores than the *PA* items, and also in comparison to the community sample, some *PA* items showed mean values which were so low, that a check for floor effects would be advisable (e.g. daring, *M* = 8.05 (*SD* = 16.44); bold, *M* = 8.43 (*SD* = 17.00).

**Table 5:** Descriptives, including Intraclass Correlation Coefficients, Intraindividual Standard Deviations, and Mean Squared Successive Differences, and of PNAI and mMDMQ items (NSSI Sample N = 51).

Item	Mean (SD)	ICC	Skewness	Kurtosis	ISD (SD)	rMSSD (SD)
At the moment I feel … PNAI items (0-100)^a^						
disgusted (angeekelt)	30.60 (32.63)	0.65	0.80	−0.54	17.84 (8.13)	21.44 (9.92)
loathing (hasserfüllt)	16.63 (25.43)	0.44	1.49	1.37	17.87 (8.24)	21.78 (9.79)
downhearted (niedergeschlagen)	37.58 (31.37)	0.42	0.44	−0.90	22.71 (7.20)	26.34 (7.98)
afraid (ängstlich)	24.98 (28.08)	0.48	0.92	−0.10	18.99 (7.64)	23.06 (9.67)
alone (allein)	33.36 (33.33)	0.49	0.64	−0.81	22.70 (8.19)	27.27 (10.28)
hostile (feindselig)	10.75 (20.17)	0.40	2.14	4.58	14.47 (8.18)	17.29 (9.12)
nervous (nervös)	29.33 (27.80)	0.39	0.78	−0.16	21.18 (5.87)	25.71 (7.76)
blameworthy (tadelnswert)	18.43 (26.16)	0.51	1.35	0.96	15.97 (9.52)	18.86 (11.45)

daring (wagemutig)	8.05 (16,44)	0.39	2.28	5.42	11.13 (7.78)	14.02 (9.83)
attentive (aufmerksam)	28.56 (26.48)	0.36	0.55	−0.63	20.77 (6.18)	26.48 (8.28)
delighted (erfreut)	17.44 (24.20)	0.34	1.37	1.26	18.03 (8.80)	22.03 (10.77)
bold (kühn)	8.43 (17,00)	0.51	2.17	4.55	9.89 (8.09)	12.66 (10.26)
happy (glücklich)	16.17 (24.16)	0.40	1.44	1.28	16.39 (9.19)	18.97 (10.66)
concentrated (konzentriert)	24.26 (24.86)	0.39	0.73	−0.31	18.54 (6.40)	23.71 (8.31)

mMDMQ items (0-100)^b^						
agitated/calm *(unruhig/ruhig)*	53.41 (24.66)	0.27	−0.20	−0.79	19.66 (6.67)	25.06 (9.24)
tense/relaxed^#^*(angespannt -entspannt*)	57.34 (23.45)	0.26	−0.40	−0.35	18.99 (6.47)	23.30 (7.60)

discontent/content^#^*(unzufrieden/zufrieden)*	54.65 (25.81)	0.31	−0.11	−0.77	20.39 (6.61)	24.64 (8.54)
unwell/well *(unwohl/wohl)*	57.79 (26.44)	0.38	−0.30	−0.66	19.74 (6.25)	24.33 (8.18)

tired/awake *(müde/wach)*	59.62 (31.26)	0.30	−0.36	−1.15	24.88 (8.21)	31.89 (11.02)
without energy/full of energy^#^*(energielos/energievoll)*	61.64 (25.81)	0.27	−0.38	−0.61	21.14 (6.55)	26.19 (8.70)

Note: PNAI = Positive and Negative Affect Items; mMDMQ = momentary Multidimensional Mood Questionnaire, SD = standard deviation, ICC = intraclass correlation, ISD = intraindividual standard deviation, rMSSD = root mean square of successive differences.

# recoded Variables (100-x), labels adapted to recoded direction.

a PNAI items were transformed form a 1-5 Likert Scale to a range of 0-100.

b mMDBF items were transformed from a 0-6 Likert Scale to a range of 0-100.

mMDMQ did depict more within-subject variance, evidenced by the *ICC* of the PNAI items ranging from 0.34 (*delighted*) to 0.65 (*disgusted*), while among the mMDMQ items, items scored between 0.26 and 0.38.

The skewness in the third column of Table 5 shows in the NSSI sample higher positive skewed distributions this time for the *PA-* and *NA* items (min = 0.44, max = 2.28). Again, the mMDMQ items scored within acceptable limits regarding normal distribution (min = −0.40, max = -0.11). The kurtosis as the description of the steepness of the distribution, in the fourth column of Table 5, showed high values, in *PA* (e.g. *daring* (5.42), *bold* (4.55), *hostile* (4.58). In the Items of the mMDMQ only *tired/awake* scored below −1 (−1.15), as the other five items ranged between −0.35 and −0.79. The *rMSSD* of PNAI items showed scores within a comparable range (min = 12.66 (*SD* = 10.26) (*bold*), max = 26.48 (SD = 8.28) (*attentive*)) compared to the mMDMQ items (min = 23.30 (*SD* = 7.60) (*tense/relaxed*), max = 26.16 (*SD* = 8.70) (*without-/full of energy*)).

### 3.2 Scales

Descriptives on scale characteristics in Table 6 show lower mean values and *SD*s in the *PA* scale (*M* = 17.15; *SD* = 15.49) and *NA* scale (*M* = 25.21; *SD* = 19.77) than in the mMDMQ scales. The small value of *PA* scale (*M* = 17.15, *SD* = 15.49), in the NSSI sample is again, suspicious of measuring floor effects same as the *NA* scale in the community sample.

**Table 6:** Means, Intraindividual Standard Deviation, Mean Squared Successive Differences, and Intra Cluster Correlation of PNAI and mMDMQ scales. (NSSI Sample, N = 51).

Scale	Mean (SD)	ICC	Skewness	Kurtosis	ISD Mean (SD)	rMSSD Mean (SD)
PNAI Scales (0-100)^a^						
negative affect	25.21 (19.77)	0.61	0.92	0.53	11.77 (4.93)	13.23 (5.65)
positive affect	17.15 (15.49)	0.46	0.87	0.19	10.88 (4.38)	12.94 (5.41)

mMDMQ Scales a (0-100)^b^						
valence	56.22 (24.08)	0.38	−0.21	−0.58	17.84 (5.96)	21.24 (7.60)
calmness	55.37 (21.58)	0.31	−0.27	−0.41	16.75 (5.82)	23.28 (8.16)
energetic arousal	60.63 (26.03)	0.32	−0.37	−0.81	20.57 (6.52)	25.58 (8.68)

Note. SD = standard deviation, ISD = intraindividual standard deviation, rMSSD = root mean square of successive differences, ICC = intraclass correlation.

a PNAI items were transformed form a 1-5 Likert Scale to a range of 0-100.

b mMDBF items were transformed from a 0-6 Likert Scale to a range of 0-100.

The ICC of the *PNAI* scales with values of 0.61 for the *PA* scale and 0.46 for *NA* scale are both higher than the scales of the mMDMQ (*ranging from* 0.31 to 0.38), evidencing more within-subject variance in the mMDMQ scales.

The *ISD* of the *NA* scale yielded a value of 11.77, which was higher than those of the *PA* scale (*ISD* = 10.88), but lower than those of the three mMDMQ scales (*ranging from* 16.75 to 20.57). The root mean square of successive differences (*rMSSD*), were lowest in the PNAI *PA* scale (*rMSSD* = 12.94; *SD* = 5.41), also low in the PNAI *NA* scale (*rMSSD* = 13.23; *SD* = 5.65), and on a higher level in the mMDMQ scales (ranging from 21.24 (*SD* = 7.60) (*valence*) to 25.58 (*SD* = 8.68) (*energetic arousal*). Thus, short-term changes in affective states could again be better depicted by the mMDMQ than by the PNAI scales.

Within-/Between Variance and Variance Proportions: Fig. 2 depicts again a three-level variance decomposition for all scales, with the percentage of variance in the left part of the figure and absolute variance in the right part. Whereas the percentage of within-subject variance seemed reasonable for all scales, the total amount of variance revealed a different picture with the *PA* scale showed the smallest value. In contrast, the *NA* scale showed an amount of variance comparable to the scales of the mMDMQ.

**Fig. 2:**
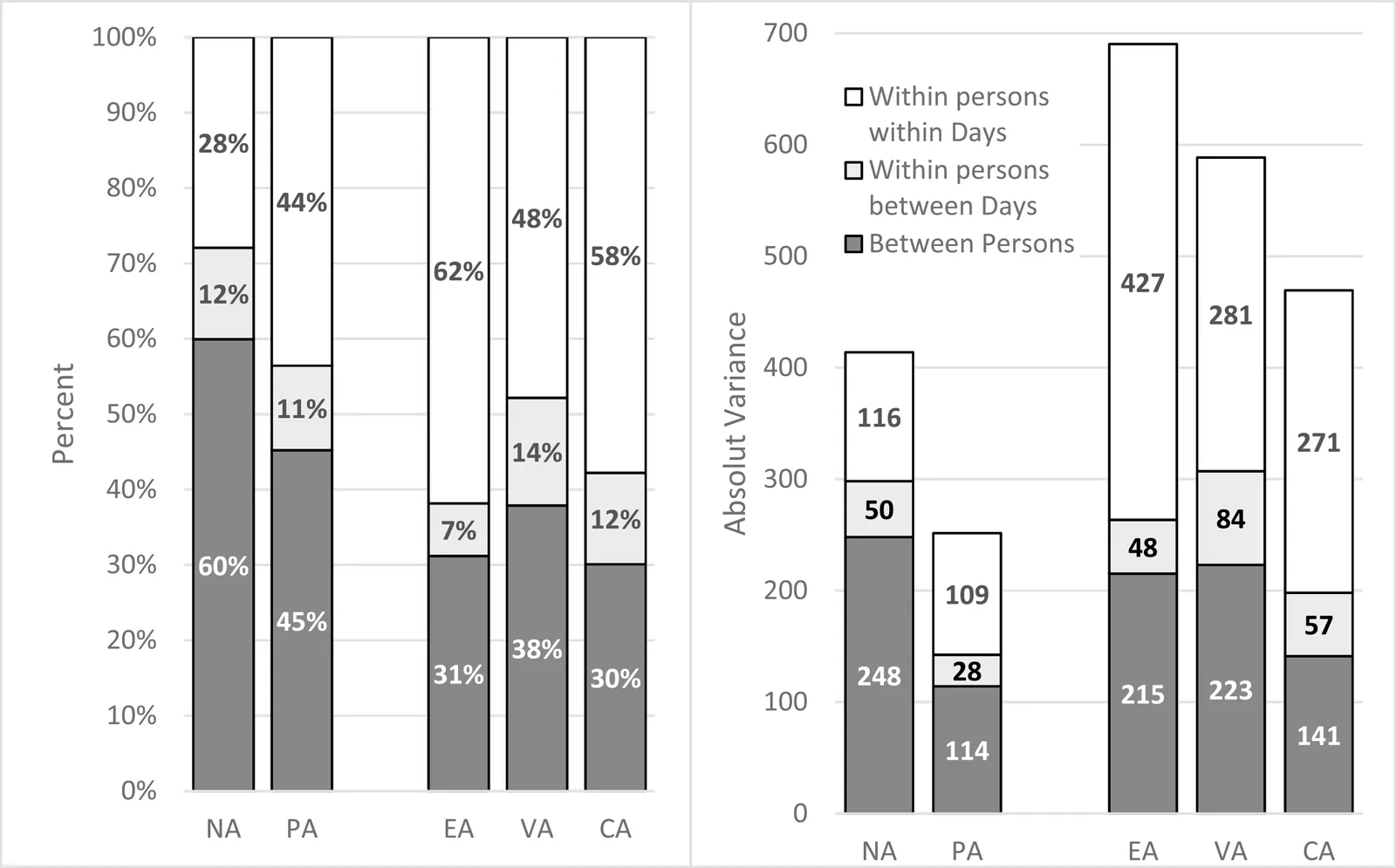
Variance: Within and Between Persons and Days. Proportion and Total Values of the PNAI and mMDMQ scales. (all Scales range from 0 to 100) (NSSI Sample N = 51) NA = negative affect, PA = positive affect, EA = energetic arousal, VA = valence, CA = calmness.

The reliability coefficient (see Table 4) as suggested by Cranford et al. (2006) showed good to excellent values for all scales. The within reliabilities are lower for all scales than the reliabilities on the between level, which showed very high values from 0.98 to 0.99. McDonalds Omega and the Spearman-Brown corrected correlations reach comparable values for all Scales.

The latent- and residual variances of all scales in the NSSI sample are shown in Table 7, which also shows a estimate for sensitivity to change as the ratio of latent within variance to latent between variance. Again, all three mMDMQ scales showed a sensitivity of change between 1.59 (*valence*) and 1.78 (*energetic arousal*). The *PA* scale of the PNAI showed a comparable sensitivity of change ratio (*PA* = 1.06), whereas the sensitivity to changes in *negative affect* was much lower (*NA* = 0.22).

**Table 7:** Latent-, Residual-Variance & Within-/Between-Variance Ratio as Sensitivity to change indicator (all scales transformed to range 0-100) (NSSI Sample N = 51).

	Latent Variance	Residual Variance	Total Variance	Latent/Total	within/between ratio
Within					
negative affect	102.92	294.98	397.90	0.26	0.22
positive affect	11.05	189.87	200.91	0.05	1.06

energetic arousal	458.53	231.25	689.77	0.66	1.78
valence	286.77	179.41	466.17	0.62	1.59
calmness	239.26	203.02	442.28	0.54	1.71

Between					
negative affect	458.34	157.13	615.46	0.74	
positive affect	10.47	85.25	95.71	0.11	

energetic arousal	257.97	33.15	291.12	0.89	
valence	179.97	23.42	203.38	0.88	
calmness	139.81	23.27	163.09	0.86	

The examination of the normal distribution of the residuals showed the highest value in skewness for *NA* there as the kurtosis scored higher in energetic arousal. All values can be found in Table S1 in the Supplement. The qq-plots of the different models in the NSSI sample are shown in the supplement (Fig. S1 and S2), with the *NA* scale again visually violating the assumption of normality significantly.

## 4 Discussion

As reporting of psychometric properties and distributional characteristics is urgently needed yet still widely neglected in e-diary research, we re-analyzed two rich datasets that assessed two different, commonly used momentary affect measures. Our goal was to examine basic distributional indices, and psychometric properties both at the item and scale level. Our results reveal a clear but nuanced pattern.

In the community sample, NA items showed pronounced floor effects, reflected in extremely low means, with small SDs and ISDs, substantial positive skew, and very high kurtosis. These characteristics persisted even after aggregation to scale level, indicating that negative affect - as assessed via unipolar adjectives - may be poorly suited to capture within-person affective dynamics in healthy samples. In contrast, PA items and all mMDMQ items displayed more favorable, near-normal distributions.

Although the PNAI or other PANAS-like questionnaires are commonly used in e-diary research, we are not aware of any studies reporting skewness and kurtosis of items and scales, although this is recommended according to recent reporting guidelines (Trull and Ebner-Priemer, 2020). As standard statistical tests often have limited capability to handle strong skewed distributions, or no within variation, information like this could help to improve the choise of the best measure for the next planed study. However, other papers did report indices that might indirectly indicate floor effects, such as means of *PA* and *NA*. Scott et al. (2020) provided a meta-analysis of momentary affect scales across different studies, including, for example, the National Study of Daily Experiences (NSDE: Almeida et al., 2009), the North Texas Heart Study (NTH: Ruiz et al., 2017) and the Work and Daily Life study (WDL: Damaske et al., 2014). All of these studies reported mean *NA* scale values comparable to our dataset (the transformed values (0-100) were: NSDE = 5.3; NTH = 7.5; WDL = 12.2), which may indicate floor effects and problematic distributions in these studies as well, especially given similar ICCs. We want to emphasize that the ICC alone does not prove sufficient within-subject variance. Small within-subject variance combined with small between-subject variance could result in ICCs that might be considered adequate, at first glance. Additional analyses are warranted, such as our analyses looking at the amount of the within-subject variance, which was critically low in the *NA* scales in the community sample. This may partly reflect the fact that the original PANAS was designed to detect between-person differences (Watson et al., 1988), not within-person fluctuations in intensive longitudinal designs.

In the clinical NSSI sample, NA items showed fewer extreme distributional problems, whereas several PA items displayed hints of floor effects. Although the PNAI items were generally not as severely skewed as the NA items in the community sample, their distributions were clearly more problematic than those of the mMDMQ. Again, this underscores that unipolar affect items - whether assessing PA or NA - carry inherent risks of floor or ceiling effects, depending on the sample. Reporting skewness and kurtosis of items and scales should become standard practice, as required by current reporting guidelines (Trull and Ebner-Priemer, 2020). As many clinical studies compare a group of healthy participants with a group of clinical cases, it is nearly inevitable that at least one group will exhibit substantial distributional distortions.

These distributional difficulties were also reflected in our multilevel analyses. For the NA scale in the community sample, multilevel models indicated that not only the *NA* scale itself was right-skewed and highly leptokurtic, but so were the residuals. In contrast, the distributions of the residuals of the *PA* scale, valence, calmness, and energetic arousal scales fulfilled basic assumptions of normality and homoscedasticity. Thus, problematic item distributions cannot simply be “fixed” through aggregation or transformation: skewed items lead to skewed residuals, potentially compromising model fit and interpretation.

Sensitivity-to-change indices corroborated these findings. The PA scale and the three mMDMQ scales demonstrated satisfactory sensitivity to within-person fluctuations, whereas the NA scale exhibited markedly low sensitivity. This is not surprising as sensitivity to change indices are based on within-subject variance.

The Spearman Brown corrected correlation coefficients of the mMDMQ in the community sample are unexpected low on the between level. With the exeption of the energetic arousal scale they are still above 0.8, but compared with the reliabity based on the work of Cranford et al. (2006) we would expect higher coefficients. Although the within- and between variance in *NA* were only small, reliability estimates were acceptable to excellent for all scales, including *NA*. When most items are scored at or near zero, inter-item correlations within time points become artificially high. Thus, reliability estimates must be interpreted cautiously and always in conjunction with distributional information.

## 5 Limitations

Debates around the conceptualization of affect - one-, two-, or three-dimensional models; distinctions between affect, mood, and emotion; and the merits of unipolar versus bipolar scaling - are longstanding and beyond the scope of this manuscript (e.g., Matthews et al., 1990; Russell, 1980; Schimmack and Rainer, 2002; Thayer, 1989). All these critical and important issues are beyond what can be dealt with in this manuscript. Nevertheless, our findings demonstrate that one widely used momentary affect measure exhibits highly problematic distributions at the item, scale, and residual level.

One might argue that the unfavorable distributions are due to the specific selection of items. This points to another persistent issue: To reduce participant burden, e-diary studies often administer only subsets of PANAS (Watson et al., 1988) or PANAS-X items (Watson and Clark, 1994), by selecting individual items from this scale (see Table 1 in Scott et al., 2020). This practice complicates comparability across studies, as psychometric properties may not generalize to arbitrary item subsets, and in some cases, scales share no overlapping items at all. We strongly recommend that future publications clearly indicate whether full scales or item subsets are used, as results can hardly be compared between different item sets. As other published datasets (NSDE: Almeida et al., 2009; WFHS: Bray et al., 2013; NTH: Ruiz et al., 2017) also show, mean values indicating floor effects, we assume that the distributions found in our dataset are not the result of an improper selection of items. As there is no accepted standard short set of PANAS or PANAS-like items for EMA studies available, the comparison of findings across studies will be impaired, unless such a standard set of items for EMA studies exist.

The same argument and counter-argument can be made for our community sample, which may not generalize. The distribution of individual variables, e.g. suspiciousness, may be significantly different in clinical studies, i.e. in samples of people with mental health problems. However, comparing healthy and clinical groups is complicated by the fact that distributional assumptions may be violated in only one group, thus undermining model comparisons. Large-scale meta-analytic efforts reporting item-, scale-, and residual-level distributions across existing datasets would be highly valuable, including analyses differentiating potential age or gender effects.

Finally, using consistent response formats across scales (i.e. using the same VAS or Likert scale) would have simplified the analyses. The transformation of the items and scales had no impact on the distribution, but the different response formats may have an effect on the variance. In addition, having only scales with at least three items would have made it possible to calculate McDonald's Omegas as the state-of-the-art reliability coefficient for all scales. Nevertheless, we believe that the main conclusions - especially those concerning problematic distributions - would not have changed.

## 6 Conclusions and recommendations

Momentary e-diary research fundamentally depends on measures that capture within-person variance. We therefore strongly recommend systematically examining and reporting within-person variance indicators such as SDs, ISDs, skewness, and kurtosis at the item level. Items exhibiting no within-person variance reflect trait-like characteristics and are unsuitable for intensive longitudinal designs. Accordingly, a specific time-based design and appropriate items must be selected to ensure within-subject variance in the phenomena of interest (Trull et al., 2015). Moreover, participants with zero variance on entire scales may unintentionaly ignored in algorithms of multilevel reliability models, as was the case for up to 30% in *NA* of individuals in the community sample. Importantly, transformations (e.g., log, gamma) may improve distributional shape but cannot create within-person variance where none exists. This aligns with recent recommendations emphasizing the importance of item selection for participant burden and data quality (Dejonckheere and Erbaş, 2021). The other reported indices, including i) proper distribution of residuals in the multilevel model, ii) sensitivity to change, and iii) reliability, are of course valuable, but cannot replace reports of within-subject variance.

Reporting distributions and psychometric properties are necessary for e-diary items in general (Trull and Ebner-Priemer, 2020). This is particularly important as many unipolar items assessing rare experiences, such as those in *NA*, may have floor effects. The mMDMQ with bipolar items, didn't show such floor effects. Nethertheless, such reporting should become established standards. As McDonald's Omega is currently the state-of-the-art approach to estimate reliability in multilevel modeling, and as this coefficient requires at least three items per scale, it should be considered that the calculation isn't possible with one- or two-item scales. On the other hand, the mMDMQ, a very well validated measurement for EMA Studies, demonstrated good distributions and psychometric properties while placing a low burden on participants.

## Declaration of generative AI and AI-assisted technologies in the manuscript preparation process

During the preparation of this work the author(s)used Deepl.com for the first draft to improve the quality of the translations. After using this tool, the author(s) reviewed and edited the content as needed and take(s) full responsibility for the content of the published article.

## Declaration of competing interest

**A.M.-L.** has received consultant fees from the Daimler and Benz Foundation, EPFL Brain Mind Institute, Fondation FondaMental, Hector II Foundation, Invisio, Janssen-Cilag GmbH, Lundbeck A/S, Lundbeckfonden, Lundbeck Neuroscience Foundation, Neurotorium, MedinCell, The LOOP Zürich, University Medical Center Utrecht, University of Washington, the Mental Wellbeing Association and the von Behring-Röntgen Foundation; speaker fees from Ärztekammer Nordrhein, Caritas, Clarivate, the German Society for Neuroscientific Assessment, Gentner Verlag, the State Medical Association Baden-Württemberg, LWL Bochum, Northwell Health, Ruhr University Bochum, Penn State University, the Society of Biological Psychiatry, the University Prague and Vitos Klinik Rheingau; and editorial or author fees from the American Association for the Advancement of Science, the European College of Neuropsychopharmacology, Servier Int. and Thieme Verlag. **U.E.-P.** reports consultancy for Boehringer Ingelheim and speaker honorarium from Angelini Pharma. The other authors declare no competing interests.

## References

[bib1] Almeida D.M., McGonagle K., King H. (2009). Assessing daily stress processes in social surveys by combining stressor exposure and salivary cortisol. Biodemogr. Soc. Biol..

[bib2] Association World Medical (2013). World Medical Association Declaration of Helsinki: ethical principles for medical research involving human subjects. JAMA.

[bib3] Bolger N., Davis A., Rafaeli E. (2003). Diary methods: capturing life as it is lived. Annu. Rev. Psychol..

[bib4] Bray J.W., Kelly E.L., Hammer L.B., Almeida D.M., Dearing J.W., King R.B., Buxton O.M. (2013). http://www.ncbi.nlm.nih.gov/books/NBK512930/.

[bib5] Cranford J.A., Shrout P.E., Iida M., Rafaeli E., Yip T., Bolger N. (2006). A procedure for evaluating sensitivity to within-person change: can mood measures in diary studies detect change reliably?. Pers. Soc. Psychol. Bull..

[bib6] Cronbach L.J., Furby L. (1970). How we should measure „change“: or should we?. Psychol. Bull..

[bib7] Csikszentmihalyi M., Larson R. (1987). Validity and reliability of the experience-sampling method. J. Nerv. Ment. Dis..

[bib8] Damaske S., Smyth J.M., Zawadzki M.J. (2014). Has work replaced home as a haven? Re-examining arlie Hochschild's time bind proposition with objective stress data. Soc. Sci. Med..

[bib9] Dejonckheere E., Erbaş Y., Germeys - Kuppens M., Myin-Germeys I., Kuppens P., Hrsg (2021). *The Open Handbook of Experience Sampling Methodology* (S. 33–69).

[bib10] Ebner-Priemer U.W., Kuo J., Kleindienst N., Welch S.S., Reisch T., Reinhard I., Lieb K., Linehan M.M., Bohus M. (2007). State affective instability in borderline personality disorder assessed by ambulatory monitoring. Psychol. Med..

[bib11] Eid M., Diener E. (1999). Intraindividual variability in affect: reliability, validity, and personality correlates. Journal of Personality and Social Psychology.

[bib12] Eisele G., Lafit G., Vachon H., Kuppens P., Houben M., Myin-Germeys I., Viechtbauer W. (2021). Affective structure, measurement invariance, and reliability across different experience sampling protocols. J. Res. Pers..

[bib13] Eisinga R., Grotenhuis M. te, Pelzer B. (2013). The reliability of a two-item scale: pearson, cronbach, or spearman-brown?. Int. J. Publ. Health.

[bib14] Fahrenberg J., Myrtek M., Pawlik K., Perrez M. (2007). Ambulantes Assessment—Verhalten im Alltagskontext erfassen. Psychol. Rundsch..

[bib15] Gable S.L., Reis H.T., Elliot A.J. (2000). Behavioral activation and inhibition in everyday life. Journal of Personality and Social Psychology.

[bib16] Gan G., Ma R., Reichert M., Giurgiu M., Ebner-Priemer U.W., Meyer-Lindenberg A., Tost H. (2021). Neural correlates of affective benefit from real-life social contact and implications for psychiatric resilience. JAMA Psychiatry.

[bib17] Geldhof G.J., Preacher K.J., Zyphur M.J. (2014). Reliability estimation in a multilevel confirmatory factor analysis framework. Psychol. Methods.

[bib18] Hepp J., Störkel L.M., Wycoff A.M., Freeman L.K., Schmahl C., Niedtfeld I. (2021). A test of the interpersonal function of non-suicidal self-injury in daily life. Behav. Res. Ther..

[bib19] Litt M.D., Cooney N.L., Morse P. (1998). Ecological momentary assessment (EMA) with treated alcoholics: methodological problems and potential solutions. Health Psychol..

[bib20] Matthews G., Jones D.M., Chamberlain A.G. (1990). Refining the measurement of mood: the UWIST mood adjective checklist. Br. J. Psychol..

[bib21] Neumann J. von, Kent R.H., Bellinson H.R., Hart B.I. (1941). The mean square successive difference. Ann. Math. Stat..

[bib22] Reichert M., Braun U., Gan G., Reinhard I., Giurgiu M., Ma R., Zang Z., Hennig O., Koch E.D., Wieland L., Schweiger J., Inta D., Hoell A., Akdeniz C., Zipf A., Ebner-Priemer U.W., Tost H., Meyer-Lindenberg A. (2020). A neural mechanism for affective well-being: subgenual cingulate cortex mediates real-life effects of nonexercise activity on energy. Sci. Adv..

[bib23] Reis H.T., Gable S.L. (2000). Handbook of Research Methods in Social and Personality Psychology.

[bib24] Rogosa D. (1988). Methodological Issues in Aging Research.

[bib25] Ruiz J.M., Taylor D.J., Uchino B.N., Smith T.W., Allison M., Ahn C., Johnson J.J., Smyth J.M. (2017). Evaluating the longitudinal risk of social vigilance on atherosclerosis: study protocol for the north Texas heart study. BMJ Open.

[bib26] Russell J.A. (1980). A circumplex model of affect. Journal of Personality and Social Psychology.

[bib27] Scherbaum C.A., Pesner E. (2019). Power analysis for multilevel research. The handbook of multilevel theory, measurement, and analysis.

[bib28] Schimmack U., Rainer R. (2002). Experiencing activation: energetic arousal and tense arousal are not mixtures of valence and activation. Emotion.

[bib29] Scollon C.N., Kim-Prieto C., Diener E. (2003). Experience sampling: promises and pitfalls, strengths and weaknesses. J. Happiness Stud.: An Interdisciplinary Forum on Subjective Well-Being.

[bib30] Scott S.B., Sliwinski M.J., Zawadzki M., Stawski R.S., Kim J., Marcusson-Clavertz D., Lanza S.T., Conroy D.E., Buxton O., Almeida D.M., Smyth J.M. (2020). A coordinated analysis of variance in affect in daily life. Assessment.

[bib31] Singer J.D., Willett J.B. (2003).

[bib32] Steyer R., Schwenkmezger P., Notz P., Eid M. (1994). Testtheoretische Analysen des Mehrdimensionalen Befindlichkeitsfragebogen (MDBF). [Theoretical analysis of a multidimensional mood questionnaire (MDBF). Diagnostica.

[bib33] Stone A.A., Shiffman S. (2002). Capturing momentary, self-report data: a proposal for reporting guidelines. Ann. Behav. Med..

[bib34] Stone A.A., Shiffman S., Schwartz J.E., Broderick J.E., Hufford M.R. (2002). Patient non-compliance with paper diaries. Br. Med. J..

[bib35] Störkel L.M., Karabatsiakis A., Hepp J., Kolassa I.-T., Schmahl C., Niedtfeld I. (2021). Salivary beta-endorphin in nonsuicidal self-injury: an ambulatory assessment study. Neuropsychopharmacology.

[bib36] Störkel L.M., Niedtfeld I., Schmahl C., Hepp J. (2023). Does self-harm have the desired effect? Comparing non-suicidal self-injury to high-urge moments in an ambulatory assessment design. Behav. Res. Ther..

[bib37] Thayer R.E. (1989).

[bib38] Tost H., Reichert M., Braun U., Reinhard I., Peters R., Lautenbach S., Hoell A., Schwarz E., Ebner-Priemer U., Zipf A., Meyer-Lindenberg A. (2019). Neural correlates of individual differences in affective benefit of real-life urban green space exposure. Nat. Neurosci..

[bib39] Trull T.J., Ebner-Priemer U.W. (2020). Ambulatory assessment in psychopathology research: a review of recommended reporting guidelines and current practices. J. Abnorm. Psychol..

[bib40] Trull T.J., Lane S.P., Koval P., Ebner-Priemer U.W. (2015). Affective dynamics in psychopathology. Emot. Rev. : Journal of the International Society for Research on Emotion.

[bib41] Watson D., Clark L.A. (1994).

[bib42] Watson D., Clark L.A., Tellegen A. (1988). Development and validation of brief measures of positive and negative affect: the PANAS scales. Journal of Personality and Social Psychology.

[bib43] Wilhelm P., Schoebi D. (2007). Assessing mood in daily life: structural validity, sensitivity to change, and reliability of a short-scale to measure three basic dimensions of mood. Eur. J. Psychol. Assess..

