# Supplementary material for: Capturing affective states in everyday life - Comparing psychometric properties of two affect measures
Source: Neurosci Appl. 2026 Sep 15;5:107026. doi: 10.1016/j.nsa.2026.107026 (PMC13631613; doi:10.1016/j.nsa.2026.107026)

Table S1. Residuals of the PNAI and mMDMQ Scales of the two Level Null Models (Intercept only) on the within- and between Level.

	Within		Between	
Scales	Skewness	Kurtosis	Skewness	Kurtosis
Community Sample (N=353)				
negative affect	-2.03	12.39	-3.04	12.35
positive affect	0.42	1.25	0.22	0.64
energetic arousal	0.46	-0.29	0.05	-0.20
valence	0.99	1.96	0.32	0.49
calmness	0.85	1.39	0.15	0.12
NSSI Sample (N=51)				
negative affect	-0.65	1.90	-0.86	0.43
positive affect	-0.72	1.25	-0.57	-0.12
energetic arousal	0.09	-0.24	0.11	-0.51
valence	-0.08	0.21	0.08	-0.32
calmness	-0.12	0.34	0.20	-0.37

Figure S1. Q-Q Plots: Residuals of the PNAI and mMDMQ Scales of the two Level Null Models (Intercept only).
(Community Sample N=353)

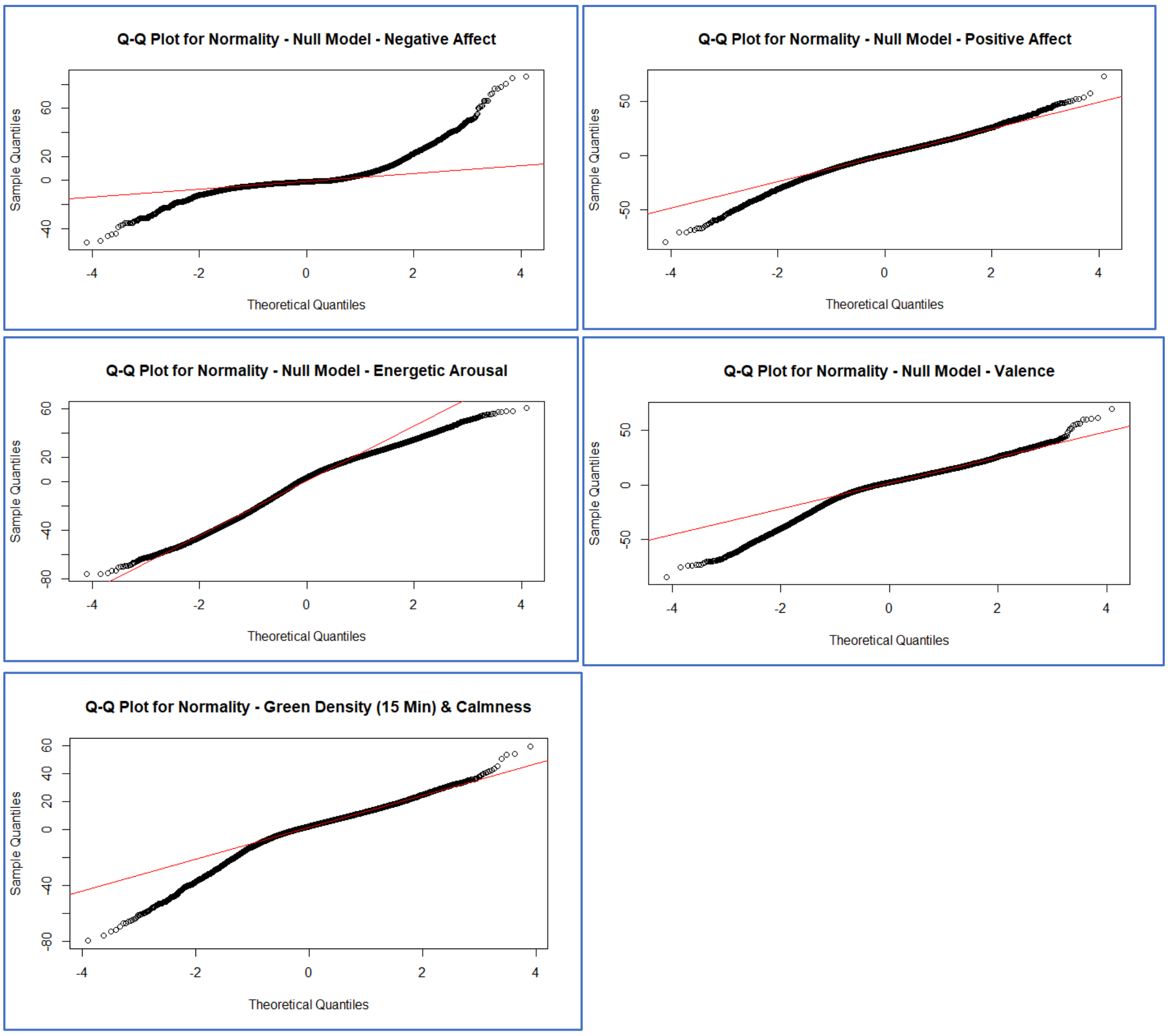

Figure S2. Q-Q Plots: Residuals of the PNAI and mMDMQ Scales of the two Level Null Models (Intercept only)
(NSSI Sample N=51) .

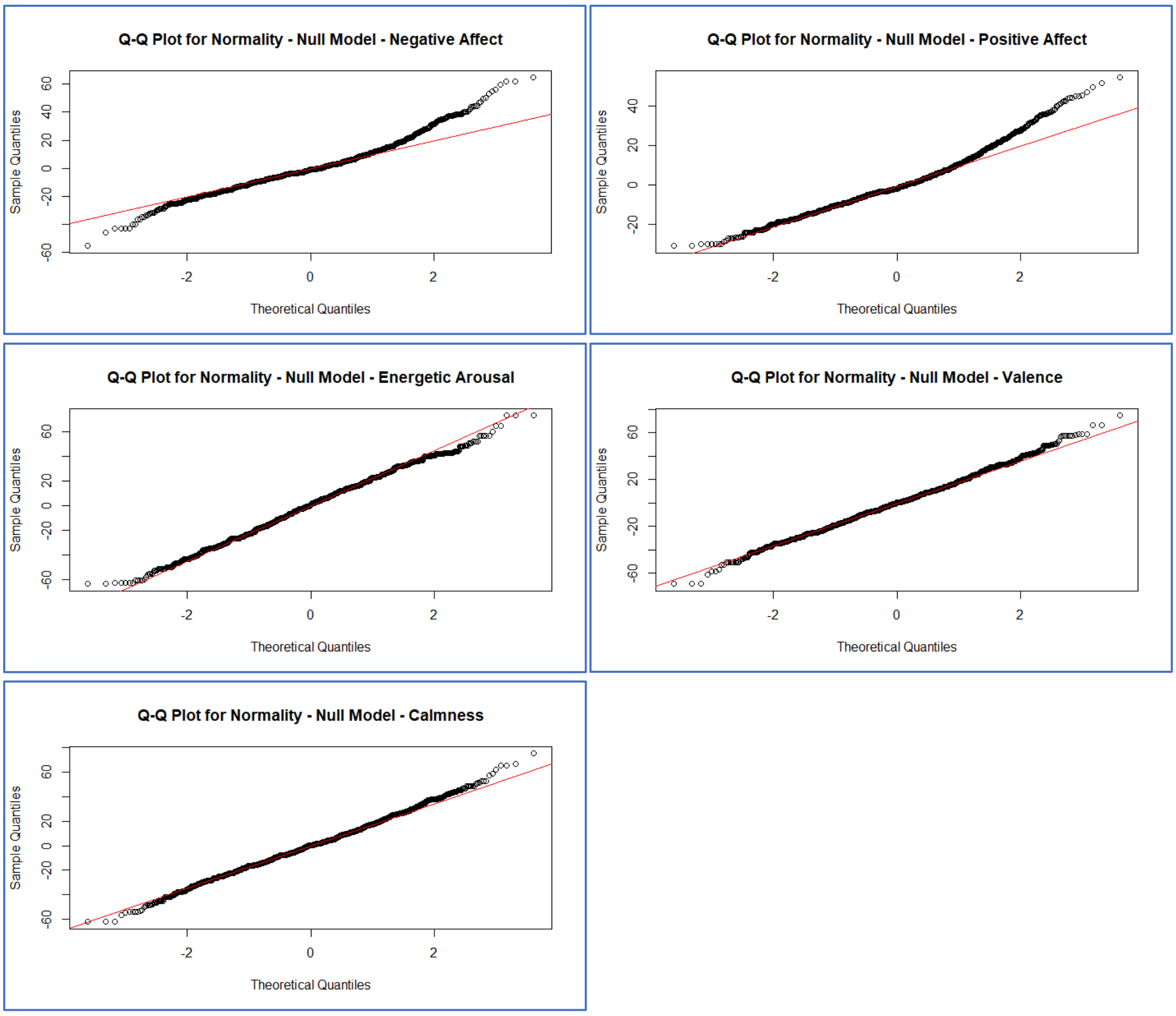

Supplement: Supplementary file 1 [file mmc1.pdf]
